# Patterns and Trends in Global Nursing Robotics Research: A Bibliometric Study

**DOI:** 10.1155/jonm/7853870

**Published:** 2025-04-04

**Authors:** Shan Zhang, Lu Liu, Tingting Peng, Shu Ding

**Affiliations:** ^1^School of Nursing, Capital Medical University, Beijing, China; ^2^Center of Cardiology, Beijing Anzhen Hospital Affiliated to Capital Medical University, Beijing, China; ^3^Nursing Department, Beijing Chaoyang Hospital Affiliated to Capital Medical University, Beijing, China

**Keywords:** aging, analysis, bibliometrics, nursing, robotics

## Abstract

**Aims:** The present study aimed to investigate the trends and research status of global nursing robot research.

**Background:** The global aging population has intensified the demand for caregiving services, highlighting the need for innovative solutions like nursing robots to address caregiver shortages and enhance healthcare efficiency. A bibliometric analysis of nursing robots from 2014 to 2024 are noteworthy but limited.

**Methods:** We searched the Web of Science database for relevant articles concerning nursing robots, published between January 1, 2014 to October 30, 2024. Data collected include: the number of publications, countries, institutions, authors, journals, reference, and keywords. CiteSpace were used to conduct the bibliometric analysis.

**Results:** The analysis included a total of 696 publications, which were produced by 165 institutions from 66 countries and involved 243 authors. The publications exhibited a generally increasing trend with fluctuations over time. The United States of America contributed the majority of articles, with 187 publications accounting for 26.87% of the total. Griffith University from Australia led the institutions with 11 publications, representing 1.58% of the overall count. The International Journal of Social Robotics was the most prolific in publishing articles on nursing robots, with 162 publications. Through the analysis of timeline graph and burst terms, we identified technology, functionality, and impact of nursing robots as research hotspots. The application of social robots in patient care and advancements in team collaboration during robot-assisted surgery are potential emerging research directions.

**Conclusions:** This study presents an overview of the nursing robot research landscape over the past decade. This research offer valuable insights and direction for future trends and research trajectories in the domain of nursing robots.

**Implications for Nursing Management:** Nursing administrators can appropriately apply nursing robots to assist in improving nursing efficiency, thereby freeing up nurses' time to focus on more complex patient care.

## 1. Introduction

The phenomenon of global population aging has been intensifying in recent years. According to the latest World Population Prospects report, there are approximately 808.9 million individuals who are aged 65 or older, representing approximately 10.0% of the global population [1]. The escalating trend of global demographic aging has led to a proportional increase in the demand for caregiving services. In numerous countries and regions, the rate of growth in the elderly demographic has outpaced the training of healthcare professionals, resulting in an increasingly pronounced shortage of caregivers [2, 3]. In addition, the scarcity of medical resources and a limited number of healthcare professionals in certain regions pose challenges in meeting the escalating demand for nursing services [2]. Therefore, the integration of nursing practice with technological advancements has become increasingly pivotal.

The emergence of nursing robots have the potential to bridge this gap, the significance of nursing robots extends beyond mere automation; they are envisioned as collaborative tools that can alleviate the burden on nurses, particularly in tasks that are repetitive or physically demanding [4, 5]. Moreover, with the rise of aging populations and the growing demand for quality healthcare, the role of nursing robots in bridging care gaps becomes ever more critical [6, 7]. The World Health Organization emphasizes the importance of innovative solutions to address the escalating needs of global health systems, and nursing robots are at the forefront of such technological interventions [8].

Nursing robots include a variety of types and functionalities, catering to the diverse care requirements of different patients [9, 10]. For instance, mobility-assistance nursebots facilitate the transfer of patients between beds and wheelchairs, while excrement management nursebots automatically detect, collect, and dispose of patients' waste [9, 11]. Daily living assistants can aid bedridden or mobility-impaired patients with activities such as eating, bathing, and dressing, and rehabilitation nursebots offer physical therapy and exercise routines [9]. The multiplicity of functions enables nursebots to comprehensively address the multifaceted nursing needs of patients.

Despite the growing interest in nursing robotics, a comprehensive bibliometric analysis of this domain remains scarce. The field of nursing robotics is complex, encompassing multidisciplinary knowledge from nursing science, engineering, artificial intelligence, and data analytics [12]. To navigate this complexity and to identify key research priorities, a systematic approach is required. Bibliometric analysis offers a methodological framework for assessing the scholarly literature on a specific topic [13]. CiteSpace, as leading visual analysis tool, has been instrumental in mapping global research trends and identifying development trend within scientific literature [14]. Therefore, our study aimed to contribute to the evidence base that informs the direction of global nursing robotics research, ensuring that it aligns with the evolving needs of healthcare and the aspirations of patient-centered care.

## 2. Materials and Methods

### 2.1. Study Design

A bibliometric study.

### 2.2. Data Source and Search Strategy

Data extraction was conducted from the Web of Science (WoS) Core Collection database using specific search criteria: “TS = (nursing OR nurse^∗^) AND TS = (robot OR robotic systems OR robotic^∗^ OR nursebot OR eldercare robot)”. The inclusion criteria were as follows: (1) studies focusing on “nursing” and “robot”; (2) publication types limited to “original articles” and “review articles”; (3) studies published in the English language; and (4) time frame for the retrieval from January 1, 2014, to October 30, 2024. The exclusion criteria were: (1) duplicated publications; and (2) publications with insufficient information to be included in the analysis.

### 2.3. Data Collection

Following the initial retrieval, two researchers screened the abstracts independently and, where necessary, the full texts of the identified literature on nursing robots, eliminating duplicates and studies unrelated to the topic. The classification of this study as nursing research is based on two key factors: (1) the primary focus of the analyzed publications on nursing-related applications of robotics, such as patient care, rehabilitation, and nursing education, and (2) the study aims to provide insights into how robotics can enhance nursing care and improve patient outcomes, aligning with the broader goals of nursing research. Discrepancies in the selection process were addressed through discussions among the authors and by consulting experts to reach a consensus. Finally, 696 articles met the inclusion criteria for the study.

### 2.4. Data Analysis

CiteSpace software 6.3.R1 was used to perform both descriptive statistics and bibliometric analyses [14]. The software was applied to count the annual output of publications pertaining to nursing robots, as well as to map the geographical distribution of these publications with the following parameter configurations: time slicing was set to an annual interval, and the network connection strength was measured using Cosine similarity. Our analysis encompassed volume and growth trend of nursing robot research. For plotting nodes of publication country, institution, author, cited author, cited journal, and references, the threshold of top 50 was set. High-frequency keywords were identified with a threshold set at the top 30, employing the minimum spanning tree method and pruning slice network for graph reduction. Keyword clustering was performed using the Log-Likelihood Ratio algorithm, with a cluster modularity value *Q* > 0.3 signifying a significant cluster structure and an average silhouette value *S* > 0.5 indicating an acceptable clustering [15]. For detecting burst keyword, the gamma value (*γ*) was set to 0.8, the minimum duration to one, and the sensitivity for citation burst detection to 2.0. The *E* value, representing the number of connection lines between nodes in the knowledge graph, indicates the diversity of cross-themes within the research field, with a higher *E* value suggesting greater diversity. The density value, calculated as the ratio of the maximum possible connections to the actual number of connections within the graph, ranges from zero to one, with higher values indicating tighter inter-node connectivity.

### 2.5. Ethical Consideration

This study is based on secondary data analysis using publicly available bibliometric databases. As such, it does not involve direct interaction with human participants or sensitive personal information.

## 3. Results

### 3.1. Publication Outputs of Nursing Robot Research

**Figure 1:**
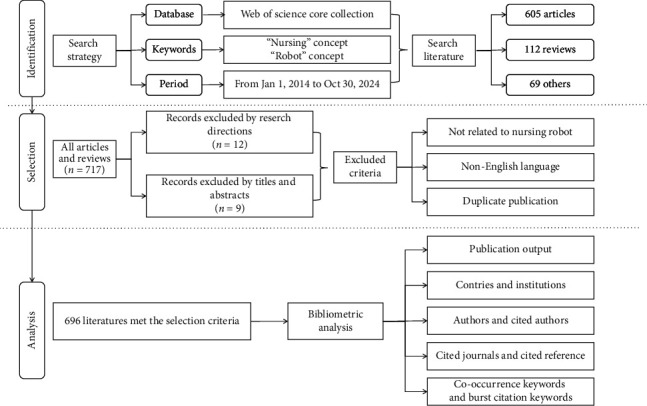
Flowchart of the literature-screening process.

A search of the WoS Core Collection database yielded a total of 786 publications. Following the literature-screening process, 696 articles were eligible for inclusion in the final analysis (Figure 1).

**Figure 2:**
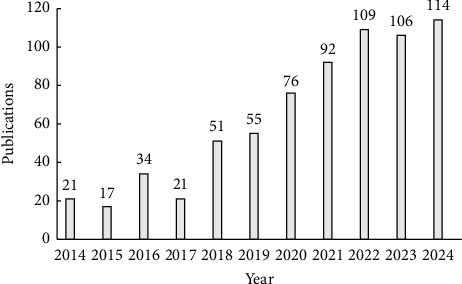
Publication output related to nursing robot research by year from 2014 to 2024.

The publication trends in nursing robots indicate an overall increasing trajectory with fluctuations. Notably, after 2022, the annual publications have consistently remained above 100, with a peak of 114 articles in 2024 (Figure 2). Between the years 2022 and 2024, a total of 329 articles were published, accounting for 47.27% of the total output. This surge in scholarly interest suggests a growing research focus on nursing robotics, leading to the field holding promising prospects for future development.

### 3.2. Publication Countries in Nursing Robots Research

A total of 66 countries participated in the publication of the articles in nursing robots. The connectivity among these countries, quantified by the connectivity index, was 321, with a density value of 0.1497. This value indicates a relatively acceptable level of collaborative effort.  Table 1 presents the top 10 contributing countries, with the United States of America leading the list with 187 publications, accounting for 26.87% of the total. China ranked second with 133 publications (19.11%), followed by Japan with 78 publications (11.21%), and Germany with 72 publications (10.34%).

### 3.3. Publication Institutions in Nursing Robots Research

A total of 165 institutions were involved in the publication of papers on nursing robot research, with 151 interinstitutional collaborations and a density of 0.0112.  Table 2 ranks the top 10 institutions by their publication output in this domain. Griffith University from Australia topped the list with 11 publications, representing 1.58% of the total. It was followed by Hebei University of Technology from China and University of Tokushima from Japan, each with 8 publications (1.15%), and Gachon University, Florida Atlantic University, and The University of Tokyo, each contributing 7 publications (1.01%).

### 3.4. Core Authors in Nursing Robots Research

A cumulative total of 243 authors have contributed to the research pertaining to nursing robots, establishing 366 collaborative links and resulting in a density of 0.0124 within this domain.  Table 3 presents the top 10 authors, ranked by their publication number. Securing the foremost position is Moley Wendy from Griffith University, Australia, with a contribution of 11 papers to the nursing robots research (1.58%).

### 3.5. Cited Authors in Nursing Robots Research

The network analysis revealed 250 cited authors with an *E* value of 1168 and a density of 0.0375.  Table 4 presents the top 10 most frequently cited authors. The top three are Moley W, with 84 publications (12.07%), affiliated with Griffith University, Australia; Robinson H, with 59 publications (8.48%); and Broadbent E, with 58 publications (8.33%), both from the University of Auckland, New Zealand.

### 3.6. Publication Journals in Nursing Robots Research

Our journal analysis identified 273 academic journals that have published research on nursing robots during the study period, indicating their significant impact in this area of study.  Table 5 ranks the top 10 most prolific journals in the field of nursing robot research. At the forefront is the International Journal of Social Robotics, with a substantial contribution of 162 articles (23.27%) and an impact factor of 3.8. Following closely are the Journal of Advanced Nursing, with 134 articles (19.25%), and the Journal of the American Medical Directors Association, with 127 articles (18.25%), both of which have made considerable contributions to the literature in nursing robot research.

### 3.7. Cited Reference in Nursing Robots Research

The nursing robots research was markedly influenced by 125 pivotal references, which helps researchers quickly grasp the research dynamics, core issues, and developmental trends in the field of nursing robot research.  Table 6 presents the 10 most frequently cited references, as determined by their citation counts in the extant literature, underscoring their essential contribution to the nursing robot research.

### 3.8. Keyword Analysis

**Figure 3:**
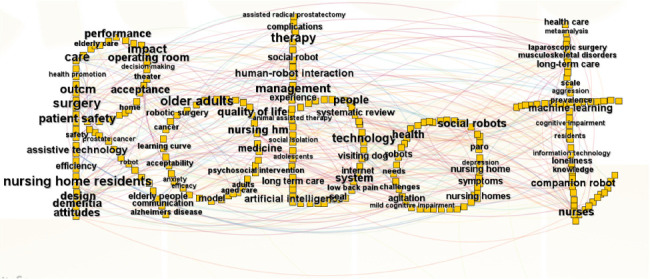
Keyword co-occurrence network.

To uncover the structural underpinnings of nursing robot research through bibliometric analysis, keywords serve as invaluable instruments, enabling the identification of emerging research frontiers. A total of 41 keywords, each appearing no fewer than 10 times, were identified in the study. We mapped the 262 keywords to generate a graphical representation (Figure 3).

Table 7 presents the top 20 keywords that are indicative of the research publications surrounding nursing robots.

**Figure 4:**
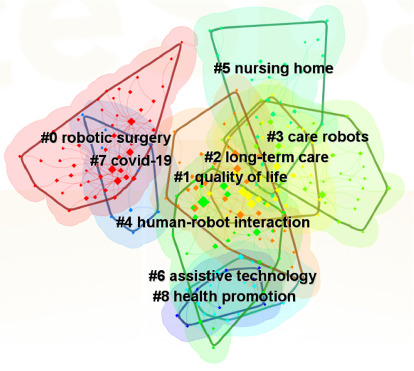
Keywords clustering graph of nursing robot research.

The results of keyword clustering analysis revealed an *S*-value of 0.8401 and a *Q*-value of 0.5631, suggesting that the clustering is both valid and reflective of the research hotspot in nursing robot research. As shown in Figure 4, each cluster is distinctly colored, providing a clear visual separation among the various groups. The keywords have been organized into nine unique thematic categories.

**Figure 5:**
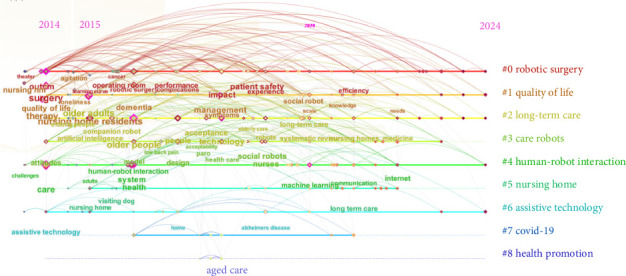
Keywords co-occurrence timeline graph of nursing robot research.

Figure 5 illustrates the distribution of nine distinct clusters within the domain of nursing robot research, each defined by its predominant keywords. Cluster 0, centered on robotic surgery, encompasses terms such as “robotic surgery”, “robotic-assisted surgery”, “experience”, “surgery”, “patient safety”, and “outcome”. Cluster 1, which focuses on quality of life, includes keywords like “nursing home residents”, “dementia”, “scale”, “loneliness”, “management”, and “therapy”. Cluster 2, concerning long-term care, features keywords such as “companion robot”, “older adults”, “technology”, “acceptance”, “artificial intelligence”, and “robots”. Cluster 3, dedicated to care robots, highlights “pain”, “social robot”, “attitudes”, “model”, “design”, “nurses”, “barriers”, and “health care”. Cluster 4, on human-robot interaction, comprises keywords such as “care”, “health”, “communication”, “nurse education”, “machine learning”, “system”, “assistive robotics”, “Alzheimer's disease”, and “emotion”. Cluster 5, pertaining to nursing homes, is characterized by “animal-assisted therapy”, “risk”, “disease”, “robotic pets”, “psychological symptoms”, and “psychiatric disorders”. Cluster 6, on assistive technology, contains keywords like “ambient assisted living”, “home”, “device”, “primary care”, and “nursing robotics”. Cluster 7, related to COVID-19, is marked by “social isolation”, “perioperative care”, and “mental health”. Lastly, Cluster 8, on health promotion, is identified by “aged care” and “health care robots.” These themes collectively represent the core areas of interest within the research on nursing robots.

Three research hotspots in nursing robot research were identified based on keywords and their clustering. The first category, pertaining to technology in nursing robot research, includes keywords such as technology, artificial intelligence, design, robot surgery, system, human-robot interaction. The keywords clustering emphasizes #0 robotic surgery, #4 human-robot interaction, and #6 assistive technology. The second category, focusing on the functions of nursing robots, is defined by terms like care, older adults, dementia, therapy, nursing home residents, and companion robot. Clustering highlights clusters #2 long-term care, #3 care robots, and #5 nursing home. The third category, which examines the impact of nursing robots, is characterized by keywords outcome, quality of life, health, impact, attitudes. The keywords clustering includes #1 quality of life, #7 COVID-19 and #8 health promotion.

**Figure 6:**
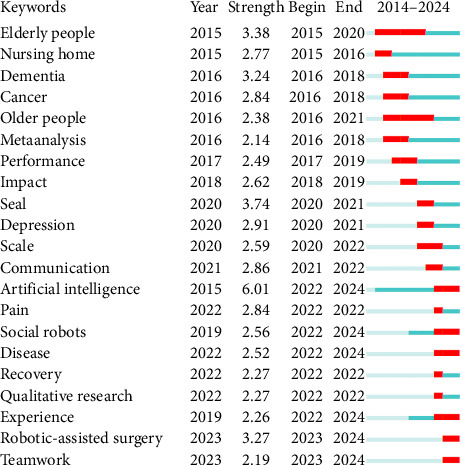
Top 19 keywords with strong citation bursts between 2014 and 2024.

Burst keywords serve as markers of emerging research interests and are reflective of the most recent developments and trends in nursing robots research.  Figure 6 presents the top 21 burst keywords identified for the period from 2014 to 2024, with the intensity of the red line indicating the period of significant attention for each keyword. The term artificial intelligence had the highest strength, followed by seal (3.74) and elderly people (3.38). Additionally, notable increases in activity were noted for keywords such as “social robots”, “disease”, “experience”, “robotic-assisted surgery”, and “teamwork”. These burst keywords suggest current research trends and may potentially develop into central themes in future studies.

## 4. Discussion

This paper leverages WoS data and employs CiteSpace software to conduct a bibliometric analysis of the nursing robotics literature, highlight research hotspots, and forecast future developmental trends. This study reveals a fluctuating yet upward trajectory in the publication trends related to nursing robot research from 2014 to 2024, indicative of the growing global interest and attention towards this field. Throughout the study period, the United States emerged as the most prolific contributor, constituting 26.87% of the total publications. Moreover, the majority of the top 10 institutions with the highest publication counts in nursing robots are located in countries with significant aging populations, such as Japan, Germany, and Korea, suggesting their pivotal role in nursing robot research. A total of 243 authors from 165 institutions across 66 countries contributed to the 696 articles on nursing robot research, showing the variability in research development among different geographical regions and emphasizing the necessity for enhanced international and inter-institutional cooperation. The International Journal of Social Robotics was identified as the most productive journal in the field of nursing robot research, which encompasses a range of articles that address the latest technologies and research findings in the field of social robotics [16, 17].

In recent years, the technology associated with nursing robot research has emerged as a prominent area of focus. As advancements in technology continue, nursing robots are increasingly capable of emulating human caregiving behaviors, providing more personalized care [18]. For example, the convergence of cutting-edge technology and medical practice in the form of robotic surgery has demonstrated significant potential and value [19, 20]. Particularly in fields such as urology, gynecology, and general surgery, robotic surgical systems offer enhanced ergonomic design, refined surgical instruments, and advanced imaging technologies [20, 21]. These advancements have substantially improved the precision and safety of surgical procedures, resulting in reduced trauma and expedited postoperative recovery for patients [22]. In terms of human-robot interaction, nursing robots must be capable of recognizing and understanding patient intentions and requirements, providing a natural communication experience [23]. To achieve these functionalities, researchers are actively exploring novel interaction methods and technologies, including speech recognition, natural language processing, and affective computing, to enhance the interactive capabilities and user experience of nursing robots [23, 24]. In the area of assistive technology, nursing robots integrate sophisticated sensors, actuators, and artificial intelligence to provide real-time patient monitoring and care, offering healthcare providers with precise decision support [25].

Our study has demonstrated that the functionality of nursing robots is a research hotspot, as evidenced by the co-occurrence and clustering analysis of keywords. Nursing robots integrate a set of advanced technologies to provide comprehensive assistance and care services to users [26]. In basic nursing care, nursing robots are capable of aiding with bathing, changing clothes, wound care, and the timely administration of medication and nutritional supplements, addressing personal hygiene and daily nursing tasks [27]. The emergence of nursing robots have offered elderly individuals, dementia patients, and nursing home residents more accessible and efficient care, thereby alleviating the strain on the nursing workforce [26]. For example, nursing robots offer environmental monitoring to prevent falls and other safety hazards, with some models even featuring emergency call functions for rapid assistance in urgent situations [28, 29]. In terms of rehabilitation training, nursing robots facilitate the indoor mobility of users with limited mobility, assisting with standing, walking, and position transfer, and can offer customized rehabilitation plans that guide users through specific exercises to promote physical recovery [9]. For dementia patients, robots can identify early risks through health monitoring and assessment, promptly alerting patients and their families to potential health issues [30]. In nursing homes, companion robots provide voice interaction and intelligent chat functions for entertainment and engagement, offering psychological comfort and reducing feelings of loneliness [7, 31]. Therefore, nursing robots can take on a substantial amount of daily nursing work, alleviating the burden on care staff and enhancing the efficiency and quality of care.

The investigation of outcomes post-implementation of nursing robot is a significant focus, encompassing improvements in patient health status, enhancements in quality of life, and increases in nursing efficiency [32]. Researchers employ experimental data and surveys to objectively evaluate the efficacy of nursing robots, using these insights to refine and optimize their design and functionalities [33]. This approach aims to offer patients more comprehensive and personalized health management services. For instance, some scholars examine the impact of robotic applications on changes in patient health behaviors, such as improvements in diet and exercise [33, 34]. By providing daily care and health monitoring, nursing robots contribute to the enhancement of patients' quality of life [32, 34]. Additionally, researchers are interested in the attitudes of patients, family members, and healthcare providers, understanding their expectations and needs regarding nursing robots to improve acceptance and satisfaction levels [35, 36]. The evolution of artificial intelligence allows nursing robots to adapt more effectively to diverse caregiving scenarios and patient needs, enhancing the precision and efficiency of nursing services [12, 31].

Burst keywords serve as predictive indicators of the developmental trajectory within the field of nursing robot research. The application of social robots in patient care is indeed one of the trends in future research. With the advent of an aging society, there is a growing demand for social and emotional support among elderly patients [37, 38]. Social robots can provide companionship and psychological support through conversation, music playback, storytelling, and other interactive methods, thereby improving patients' mental health [37]. As these robots interact with patients, they accumulate care experience and gain insights into patients' preferences and needs, future studies can further optimize their interaction strategies. Innovative team collaboration models in robot-assisted surgery represent another trend in future research. Robot-assisted surgery involves knowledge and technologies from multiple disciplines, including mechanical engineering, electronics, computer science, and medicine [39]. As interdisciplinary collaboration deepens, these team collaboration models will continue to evolve. For instance, robots can take on more surgical operation tasks and patient monitoring, alleviating the burden on medical staff [39, 40]. With their high precision, minimal trauma, and rapid recovery advantages, nursing robots are gradually becoming widely applied in the medical field [41]. In the future, nursing robots are expected to integrate more deeply into patient care, becoming an indispensable part of patients' lives.

### 4.1. Limitations and Future Research

It is essential to acknowledge the limitations present in our study. Although the WoS database offers an extensive multidisciplinary academic literature abstract index, our exclusive criteria may have resulted in the exclusion of important findings from other sources. Additionally, our research team determined the data retrieval process based on a literature review, which could have introduced biased data sets. To address these limitations in future work, we plan to expand our data sources and improve our search strategy to increase the reliability of the results.

## 5. Conclusions

This study conducted a bibliometric analysis of nursing robot research from 2014 to 2024, utilizing CiteSpace software and data sourced from the WoS. The analysis revealed the current hotspots and global trends within the domain of nursing robot research. It showed an upward trajectory in the publication of articles related to nursing robots, with the USA emerging as the preeminent contributor to global research in this area. The study also highlighted the collaborative networks that need to be improved among countries, institutions, and prolific authors. Furthermore, it identified technology, functionality, and the impact of nursing robots as central research hotspots, suggesting that the application of social robots in patient care and the evolution of team collaboration in robot-assisted surgery could represent forthcoming areas of interest and research directions in this field.

### 5.1. Implications for Nursing Management

This analysis included the number of publications, contributing countries, institutions, authors, journals, references, and keywords. The contribution of this paper was to ascertain the current state of research in nursing robotics and to identify the hotspots and developmental trends. The introduction of nursing robots offers a new perspective for nursing management. They can take over tasks that are highly repetitive and labor-intensive, thereby freeing up nursing resources to focus on providing more complex and personalized care services. This not only improves work efficiency but also helps to enhance the quality of patient care, while opening up new paths for the professional development of nurses. In the future, nursing management will need to consider how to integrate robotic technology, optimize workflow processes, and ensure the humanization and safety of patient care.

## Figures and Tables

**Table 1:** Top 10 countries by number of publications.

Rank	Country	Publications, *n* (%)	Centrality
1	United States of America	187 (26.87)	0.28
2	China	133 (19.11)	0.04
3	Japan	78 (11.21)	0.04
4	Germany	72 (10.34)	0.00
5	Australia	45 (6.47)	0.23
6	England	41 (5.89)	0.30
7	South Korea	35 (5.03)	0.02
8	Italy	30 (4.31)	0.13
9	Netherland	28 (4.02)	0.03
10	France	24 (3.45)	0.03

**Table 2:** Top 10 institutions by number of publications.

Rank	Institutions	Country	Publications, *n* (%)
1	Griffith University	Australia	11 (1.58)
2	Hebei University of Technology	China	8 (1.15)
3	University of Tokushima	Japan	8 (1.15)
4	Gachon University	Korea	7 (1.01)
5	Florida Atlantic University	USA	7 (1.01)
6	The University of Tokyo	Japan	7 (1.01)
7	Bond University	Australia	6 (0.86)
8	Seoul National University	Korea	6 (0.86)
9	Kochi University	Japan	6 (0.86)
10	Technical University of Munich	Germany	5 (0.72)

**Table 3:** Top 10 authors who published nursing robot research.

Rank	Author	Publications, *n* (%)
1	Moley, Wendy	11 (1.58)
2	Guo,Shijie	9 (1.29)
3	Jones, Cindy	8 (1.15)
4	Betriana, Feni	7 (1.01)
5	Locsin, Rozzano C	6 (0.86)
6	Tanioka, Tetsuya	6 (0.86)
7	Osaka, Kyoko	5 (0.72)
8	Kim, Jeongeun	4 (0.57)
9	Lee, Hyeongsuk	4 (0.57)
10	Ota, Jun	4 (0.57)

**Table 4:** Top 10 cited authors who published nursing robot research.

Rank	Author	Publications, *n* (%)	Centrality
1	Moley W	84 (12.07)	0.15
2	Robinson H	59 (8.48)	0.07
3	Broadbent E	58 (8.33)	0.18
4	Wada K	51 (7.33)	0.08
5	Joranson N	43 (6.18)	0.03
6	Pu LH	43 (6.18)	0.12
7	Shibata T	37 (5.32)	0.14
8	Heerink M	35 (5.03)	0.04
9	Bemelmans R	32 (4.60)	0.07
10	Cohen-Mansfield J	26 (3.74)	0.05

**Table 5:** Top 10 productive journals in nursing robot research.

Rank	Journal	Publications, *n* (%)	Impact factor (journal citation reports 2023)
1	International Journal of Social Robotics	162 (23.27)	3.8
2	Journal of Advanced Nursing	134 (19.25)	3.8
3	Journal of The American Medical Directors Association	127 (18.25)	4.2
4	Plos One	113 (16.24)	2.9
5	Journal of Clinical Nursing	106 (15.23)	3.2
6	Robotics and Autonomous Systems	104 (14.94)	4.3
7	BMJ Open	95 (13.65)	2.4
8	Surgical Endoscopy and Other Interventional Techniques	88 (12.64)	2.4
9	Sensors	81 (11.64)	3.4
10	BMJ-British Medical Journal	79 (11.35)	93.7

**Table 6:** Top 10 cited reference by the nursing robot research.

Rank	Title (author)	Year	Journal	Count	Centrality
1	Use of a robotic seal as a therapeutic tool to improve dementia symptoms: a cluster-randomized controlled trial (Moyle W)	2017	Journal of the American Medical Directors Association	29	0.19
2	The utilization of robotic pets in dementia care (Petersen S)	2017	Journal of Alzheimers Disease	20	0.06
3	Scoping review on the use of socially assistive robot technology in elderly care (Abdi J)	2018	BMJ Open	20	0.13
4	Effects on symptoms of agitation and depression in persons with dementia participating in robot-assisted activity: a cluster-randomized controlled trial (Joranson N)	2015	Journal of The American Medical Directors Association	19	0.39
5	A pilot randomized trial of a companion robot for people with dementia living in the community (Liang A)	2017	Journal of The American Medical Directors Association	18	0.06
6	The benefits of and barriers to using a social robot PARO in care settings: a scoping review (Hung LL)	2019	BMC Geriatrics	17	0.05
7	The effectiveness of social robots for older adults: a systematic review and meta-analysis of randomized controlled studies (Pu LH)	2019	Gerontologist	17	0.22
8	Change in quality of life in older people with dementia participating in Paro-activity: a cluster-randomized controlled trial (Joranson N)	2016	Journal of Advanced Nursing	15	0.04
9	Care staff perceptions of a social robot called Paro and a look-alike Plush Toy: a descriptive qualitative approach (Moyle W)	2018	Aging & Mental Health	14	0.14
10	The PRISMA 2020 statement: an updated guideline for reporting systematic reviews (Page MJ)	2021	BMJ-British Medical Journal	13	0.02

**Table 7:** Top 20 keywords that published papers on nursing robots.

Rank	Keywords	Count	Centrality
1	Care	74	0.15
2	Artificial intelligence	48	0.04
3	Older adults	45	0.17
4	People	41	0.05
5	Technology	37	0.11
6	Dementia	35	0.09
7	Quality of life	35	0.18
8	Robot surgery	33	0.04
9	Health	31	0.16
10	Therapy	31	0.18
11	Surgery	31	0.23
12	Design	30	0.10
13	Impact	29	0.05
14	System	28	0.10
15	Nursing home residents	28	0.19
16	Companion robot	26	0.04
17	Outcome	25	0.12
18	Human-robot interaction	23	0.09
19	Attitudes	23	0.08
20	Nurses	19	0.09

## Data Availability

The data that support the findings of this study are available from the corresponding author upon reasonable request.
